# A Regional and Comparative Materia Medica

**Published:** 1895-06

**Authors:** 


### A Regional and Comparative Materia Medica. By John Gilmore Malcolm, M. D., and Oscar Burnham Moss, M. D. Chicago: Malcolm & Moss, publishers. 1895.

This volume is the ordinary materia medica of the homœopathic school, arranged on the regional plan. That is to say, instead of all the symptoms of any one remedy being arranged under the name of that remedy, and divided off into groups, according to the region, as we find them in all the usual works on materia medica, in the volume under review the whole work is divided into chapters, each chapter named for one of the regions, and then under the chapter heading the remedies are arranged in alphabetical order; and only the symptoms relating to that region are placed under the name of the remedy.

Thus under the chapter entitled Mind, we have all the remedies which have mental symptoms, arranged in alphabebetical order, with their indications given with scrupulous fidelity. At the end of the chapter we find a repertory to these indications.

Turning now to the chapter entitled Inner Head, we find the complete symptomatology of the head with all the various forms of headache, each given under its appropriate remedy, and these remedies all in alphabetical order. At the end of the chapter we find, as before, a repertory to the chapter, so that we can find any symptom desired. This arrangement runs all through the book. The chapters, as before stated, are simply the names of the regions of the body just as we find them, in the ordinary materia medica.

At the close of the book we find two general and highly important chapters, one entitled Aggravations, and the other Ameliorations. Conditions forming so important a part of the homœopathic prescription, it is likely that these chapters will be consulted very largely by the earnest seeker after the simillimum, and their pages will show much "thumbing." Finally, we have an alphabetical index to the repertories, so that any region may be quickly found and the indications properly examined.

Speaking of this index in the preface, the authors say: "It is another important aid in finding in the most direct manner exactly what is wanted, thus again illustrating the grand object of this work. Moreover, we venture to hope that the brief definitions of numerous technical terms in the 'Index to Repertories' will be of sufficient help to the student to justify the space we have devoted to them."

In reference to the sources from which the symptomatology of this book was derived, the authors say: "Our authorities, in respect to matter, have been the great masters from Hahnemann to Hering and Allen, not excluding the latest faithful exponent of the homœopathic materia medica. We have aimed to present a faithful report in every line; and strengthened by fraternal criticism and continual research, we pledge ourselves to supply to our utmost, in future editions, the deficiencies of the present work."

From the foregoing review, it will be apparent that this new work on materia medica is a valuable addition to the facilities for finding the simillimum, and should be possessed by every homœopathic prescriber.

